# Real-World Assessment of the Efficacy of Computer-Assisted Diagnosis in Colonoscopy: A Single Institution Cohort Study in Singapore

**DOI:** 10.1016/j.mcpdig.2024.10.002

**Published:** 2024-10-26

**Authors:** Gabrielle E. Koh, Brittany Ng, Ronja M.B. Lagström, Fung-Joon Foo, Shuen-Ern Chin, Fang-Ting Wan, Juinn Huar Kam, Baldwin Yeung, Clarence Kwan, Cesare Hassan, Ismail Gögenur, Frederick H. Koh

**Affiliations:** a Department of Surgery, Sengkang General Hospital, Singapore, Singapore; b Colorectal Service, Department of Surgery, Sengkang General Hospital, Singapore, Singapore; c Department of Gastroenterology, Sengkang General Hospital, Singapore, Singapore; d Center for Surgical Science, Department of Surgery, Zealand University Hospital, Koege, Denmark; e Department of Biomedical Sciences, Humanitas University, Rozzano, Milan, Italy; f Endoscopy Unit, Humanitas Clinical and Research Center IRCCS, Rozzano, Milan, Italy; g Department of Surgery, Duke-National University of Singapore (NUS) Medical School, National University of Singapore, Singapore, Singapore; h Department of Surgery, Lee Kong Chian School of Medicine, Nanyang Technological University, Singapore, Singapore

## Abstract

**Objective:**

To review the efficacy and accuracy of the GI Genius Intelligent Endoscopy Module Computer-Assisted Diagnosis (CADx) program in colonic adenoma detection and real-time polyp characterization.

**Patients and Methods:**

Colonoscopy remains the gold standard in colonic screening and evaluation. The incorporation of artificial intelligence (AI) technology therefore allows for optimized endoscopic performance. However, validation of most CADx programs with real-world data remains scarce. This prospective cohort study was conducted within a single Singaporean institution between April 1, 2023 and December 31, 2023. Videos of all AI-enabled colonoscopies were reviewed with polyp-by-polyp analysis performed. Real-time polyp characterization predictions after sustained polyp detection were compared against final histology results to assess the accuracy of the CADx system at colonic adenoma identification.

**Results:**

A total of 808 videos of CADx colonoscopies were reviewed. Out of the 781 polypectomies performed, 543 (69.5%) and 222 (28.4%) were adenomas and non-adenomas on final histology, respectively. Overall, GI Genius correctly characterized adenomas with 89.4% sensitivity, 61.7% specificity, a positive predictive value of 85.4%, a negative predictive value of 69.8%, and 81.5% accuracy. The negative predictive value for rectosigmoid lesions (80.3%) was notably higher than for colonic lesions (54.2%), attributed to the increased prevalence of hyperplastic rectosigmoid polyps (11.4%) vs other colonic regions (5.4%).

**Conclusion:**

Computer-Assisted Diagnosis is therefore a promising adjunct in colonoscopy with substantial clinical implications. Accurate identification of low-risk non-adenomatous polyps encourages the adoption of “resect-and-discard” strategies. However, further calibration of AI systems is needed before the acceptance of such strategies as the new standard of care.

Colonoscopy is the international gold standard for colonic screening and assessment,^1^^,^^2^ being the modality of choice in achieving histological diagnosis of colonic lesions and to perform therapeutic interventions such as polypectomies.^3^ Given the well-established evolutionary pathway of most colorectal tumor carcinogenesis,^4^ timely removal of pre-malignant lesions halts progression along the adenoma-carcinoma sequence and serrated pathway.^3^, ^4^, ^5^ This is associated with an 80% and 60% reduction in incidence and mortality from colorectal cancer, respectively.^6^^,^^7^

Endoscopy therefore is an important skill for interventionists to acquire and master.^8^ It demands successful real-time toggling of scope controls to obtain adequate mucosal exposure and meticulous inspection of mucosal surfaces for lesions and ensuring patient safety and comfort. This requires substantial experience to master and maintain consistency.^9^ As such, the integration of artificial intelligence (AI) deep learning algorithms such as computer-assisted detection in colonoscopy aims to optimize polyp detection,^10^^,^^11^ reducing the mental burden on the endoscopist.^12^ The incorporation of AI into conventional endoscopy has resulted in increased adenoma detection rates (ADRs) and adenoma per colonoscopy numbers, which may in turn be associated with a decreased risk of interval colorectal malignancy.^13^^,^^14^

Advancements in AI technology have opened doors to not only real-time detection but also optical characterization of polyps during colonoscopy. Computer-Assisted Diagnosis (CADx) programs are built on the foundation of computer-assisted detection, boasting value-added capabilities in characterizing detected colonic lesions by differentiating adenomatous polyps from non-adenomatous counterparts. This may potentially influence the endoscopist’s choice of intervention, allowing for reduced resections of non-neoplastic polyps (“leave in situ” strategy) or reduced sending of resected specimens for histological analysis (“resect-and-discard” strategy), of which both strategies may lead to an improvement in overall long-term cost-effectiveness.^15^^,^^16^ Existing real-world data on the efficacy of CADx is promising, but further research and validation are required to support the incorporation of the aforementioned “leave in situ” or “resect-and-discard” strategies into mainstream health care services as the new standard of care.^17^

Our study aims to review the accuracy of the CADx function of the GI Genius Intelligent Endoscopy Module in the identification and characterization of colonic adenomas. This aims to satisfy the unmet clinical need to evaluate this CADx program for its suitability to aid in “leave in situ” or “resect-and-discard” strategies.

## Patients and Methods

### Study Design

A prospective cohort study was conducted within a single institution in Singapore from April 2023 to December 2023 in accordance with the Strengthening the Reporting of Observational Studies in Epidemiology guidelines. This was performed as part of the CO-PILOT study (Clinicaltrials.gov ID: NCT05822895). Endoscopists in this institution utilized real-time AI-assisted colonoscopy equipment, the GI Genius Intelligent Endoscopy Module with the CADx feature, in conjunction with the unit’s pre-existing Olympus EVIS EXERA III 190 Video endoscopy system (Olympus Medical System Corp.). Colonoscopies were performed by accredited trainees and specialists in the Endoscopy unit as per the institution’s standard of care. All colonoscopies utilizing AI-assisted technology were recorded and reviewed by 2 independent authors not involved in performing these colonoscopies. Real-time polyp characterization predictions were compared with the corresponding histopathology report of the polypectomy specimens. Polypectomy was defined as the complete removal of a polyp either in whole or piecemeal fashion.

### CADx Program

The CADx program utilized in the institution was the GI Genius Intelligent Endoscopy Module. On sustained identification of a mucosal finding by the CADx system, a green box encircles the identified lesion. Real-time polyp characterization yields 3 possible polyp characterizations—“adenoma,” “nonadenoma,” or “no prediction,” as reported in the Figure. This is presented as floating labels adjacent to the superimposed detection box. “Adenoma” was defined as a polyp of any adenomatous histology, regardless of dysplasia grade. “Nonadenoma” was defined to include nonpolyp lesions (polypoid colonic mucosa and lymphoid hyperplasia) as well as all other non-adenomatous polyp histology (sessile serrated, hyperplastic, and inflammatory). Mucosal lesions, which yielded sustained detection but could not be characterized confidently using the AI system as either “adenoma” or “nonadenoma,” were resultantly labeled as “no prediction.”

**Figure:**
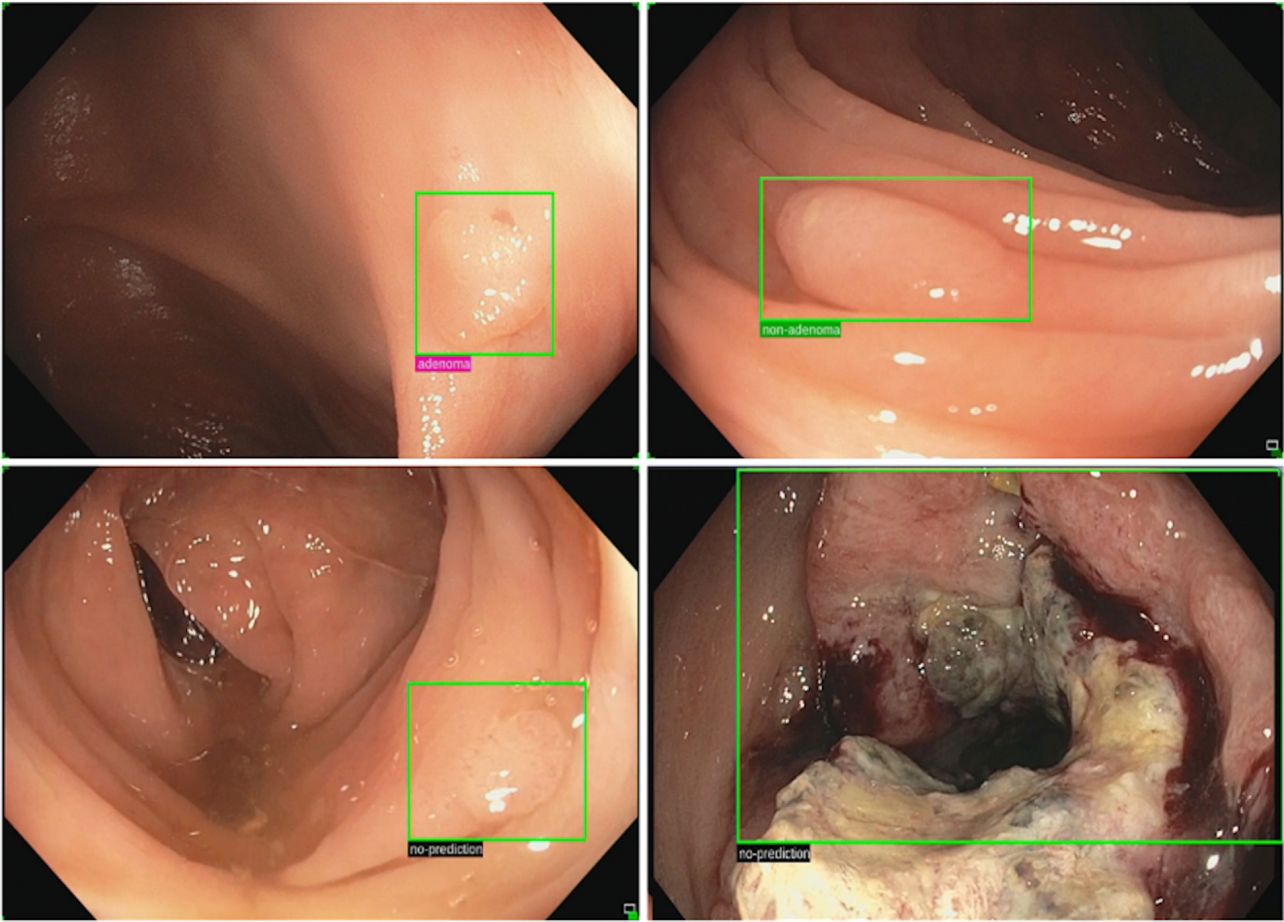
Examples of real-time GI Genius Computer-Assisted Diagnosis (CADx) polyp characterizations. Each CADx-identified mucosal lesion is encircled within a green box, accompanied by a label of its predicted characterization: “adenoma,” “nonadenoma,” or “no prediction.” The top left and right images depict the CADx characterizations of a mucosal lesion as “adenoma” and “nonadenoma,” respectively. The bottom left and right images depict mucosal lesions, which could not be conclusively characterized by the CADx as either an “adenoma” or “nonadenoma,” resulting in a “no prediction” label.

### Statistical Analyses

Polyp characterization predictions made using the CADx were compared with corresponding individual polyp histology results to assess the accuracy of the real-time polyp prediction algorithm. “No prediction” characterizations on the CADx and histology-proven malignant neoplasms were omitted from further data analysis.

Confusion matrices were plotted to attain true positive, false positive, true negative, and false negative values for CADx predictions. Computer-Assisted Diagnosis performance was evaluated on the basis of sensitivity, specificity, positive predictive value, and negative predictive value (NPV) of the characterization of adenomatous polyps by the AI algorithm. A true positive was defined as a lesion for which the CADx prediction of “adenoma” was in concordance with an adenomatous lesion on final histopathological analysis. Tubullovillous adenomas with features of both low-grade and high-grade dysplasia on final histology were included. A true negative was defined as a lesion that yielded a “nonadenoma” CADx prediction with a final histopathological report of a non-adenomatous lesion. This was taken to include all other lesions apart from colonic adenomas—namely hyperplastic polyps, sessile serrated lesions (SSLs), and polypoid colonic mucosa. Conversely, a false positive was defined as a lesion with an “adenoma” CADx prediction but yielded non-adenomatous histology, as previously defined. The false negative was similarly defined as a lesion predicted as “nonadenoma” using CADx but with final adenomatous histology.

Subgroup analysis was performed to compare the aforementioned performance markers between polyp locations. Colonic polyps were defined as polyps located in the caecum, ascending colon, hepatic flexure, transverse colon, splenic flexure, and descending colon. Rectosigmoid polyps were defined as polyps located in the sigmoid colon and rectum. This anatomical division of polyp location was adopted in keeping with American Society of Gastrointestinal Endoscopy guidelines on “diagnose-and-leave” strategies for clear NPV comparison.

Overall polyp detection rate, ADR, and adenoma per positive colonoscopy were also tabulated. Polyp detection rate was defined as the percentage of colonoscopies performed where one or more polyps were detected. Adenoma detection rate was defined as the percentage of colonoscopies performed where one or more adenomas were detected. Adenoma per positive colonoscopy was defined as the ratio of the total number of adenomas detected to the total number of colonoscopies in which at least 1 adenoma was detected. All statistical analyses were performed using IBM SPSS Statistics 28 software.

## Results

Over the study period of 9 months, 808 full videos of CADx colonoscopies were reviewed. In total, 820 CADx characterizations were generated, of which 479 (58.4%) were labeled “adenoma,” 173 (21.1%) labeled “nonadenoma.” and 168 (20.5%) labeled “no prediction” (Table 1). In total, 39 of these CADx characterizations did not result in eventual polypectomy by the endoscopist—comprising 3 predicted “adenoma,” 24 predicted “nonadenoma” and 12 “nil prediction” characterizations. The final decision for polypectomy was left to the discretion of the individual endoscopist to mirror real-life diagnostic and intervention practices before the introduction of the CADx system. Unpaired data sets such as CADx predictions without histology and polypectomized lesions without prior CADx characterization were excluded from further analysis as a CADx prediction with a corresponding histopathological result of the same lesion is required to assess algorithm accuracy.

**Table 1:** Tabulation of GI Genius Computer-Assisted Diagnosis Characterizations

GI Genius CADx characterization	Overall (%)	CADx^a^ characterizations without polypectomy
Adenoma	479 (58.4)	3
Nonadenoma	173 (21.1)	24
“No prediction”^a^	168 (20.5)	12
Total characterizations	820 (100)	39

a “No prediction” Computer-Assisted Diagnosis (CADx) characterizations and CADx characterizations without polypectomy were excluded from further analysis.

A total of 781 polypectomies were performed, of which 543 (69.5%) and 222 (28.4%) were adenomas and non-adenomas on final histology, respectively (Table 2). Of the non-adenomatous polyps, 26 (3.3%) were SSL, 115 (14.7%) were hyperplastic polyps, and 81 (10.4%) were non-polyps (colonic mucosa, lymphoid hyperplasia, and lipoma). Of the 781 polypectomies performed, 508 (65.0%) were for colonic polyps, whereas the remaining 273 (35.0%) were for rectosigmoid polyps. Final histopathology of the colonic polyps yielded a distribution of 402 (51.5%) adenomas, 13 (1.7%) SSL, and 37 (5.4%) hyperplastic polyps. This is in comparison to 141 (18.1%) adenomas, 13 (1.7%) SSL, and 78 (11.4%) hyperplastic polyps in the rectosigmoid region (Table 2). Of note, the disease prevalence of hyperplastic polyps in the rectosigmoid region was higher at 9.99% compared with 4.74% in the rest of the colon, consistent with existing literature.^18^

**Table 2:** Tabulation of Final Histopathology of Removed Specimens, With Subgroup Analysis by Location–Colonic vs Rectosigmoid^a^

Final histopathology	Overall (%)	Colonic (%)	Rectosigmoid (%)	Polypectomies without preceding CADx characterization
Adenoma	543 (69.5)	402 (51.5)	141 (18.1)	8
Sessile serrated lesion	26 (3.33)	13 (1.66)	13 (1.66)	0
Hyperplastic polyp	115 (14.7)	37 (4.74)	78 (9.99)	1
Malignant neoplasm	16 (2.05)^b^	4 (0.51)	12 (1.54)	…
Non-polyps(colonic mucosa, lymphoid hyperplasia, and lipoma)	81 (10.4)	52 (6.66)	29 (3.71)	0
Total polypectomies	781 (100)^c^	508 (65.0)	273 (35.0)	9

a CADx, Computer-Assisted Diagnosis.

b Malignant neoplasms were excluded from further analysis.

c Lost polyp specimens were excluded from the total polypectomy count.

In total, 16 specimens returned as malignant neoplasms, 4 of which were colonic malignancies and 12 rectosigmoid malignancies. Moreover, all 16 of these malignant specimens were noted to have yielded “no prediction” CADx characterizations. These neoplasms were therefore excluded from further analysis.

Of the 781 polypectomies performed, 9 were decisively performed by the endoscopists before preceding CADx characterization could be generated. In total, 8 returned with adenomatous histology and 1 as a hyperplastic polyp. These too were omitted from further analysis on the basis of unpaired data.

Of the 26 SSLs identified in this study, 2 of these lesions had characteristics of dysplasia identified on histological analysis. One sessile serrated lesions with dysplasia (SSL-d) lesion yielded a CADx prediction of “adenoma,” although the other SSL-d lesion was predicted as “nonadenoma.”

The comparison of CADx predictions against the corresponding final histopathology of the removed specimens yielded an overall true positive of 403, a true negative of 99, a false positive of 48, and a false negative of 66 (Table 3). Overall, the GI Genius was able to correctly characterize adenomas with a sensitivity of 89.4%, specificity of 61.7%, positive predictive value of 85.4%, NPV of 69.8%, and accuracy of 81.5%. Of note, the NPV of the CADx for rectosigmoid lesions was 80.3%, substantially higher than the NPV of 54.2% for colonic lesions. A polyp detection rate of 44.9%, ADR of 36.4%, and adenoma per positive colonoscopy of 1.50 were obtained (Table 4).

**Table 3:** Polyp-by-Polyp Comparison of GI Genius Computer-Assisted Diagnosis Polyp Characterizations Against Final Histopathology of Removed Polyp Specimens

GI Genius CADx characterizations	Final histopathology of removed polyp specimens					
	Adenoma			Nonadenoma		
Types of output	Overall	Colonic	Rectosigmoid	Overall	Colonic	Rectosigmoid
Adenoma	403	300	103	66	33	33
Nonadenoma	48	33	15	99	38	60

CADx, Computer-Assisted Diagnosis.

**Table 4:** Evaluation of the Overall Performance of GI Genius Computer-Assisted Diagnosis Algorithm

Evaluated parameter	Performance of GI Genius CADx algorithm		
	Overall	Colonic	Rectosigmoid
Sensitivity	89.4%	90.1%	87.3%
Specificity	61.7%	54.2%	64.9%
Positive predictive value	85.4%	90.1%	75.7%
Negative predictive value	69.8%	54.2%	80.3%
Accuracy	81.5%	83.7%	77.4%
Polyp detection rate	44.9%	…	…
Adenoma detection rate	36.4%	…	…
Adenoma per positive colonoscopy	1.50	…	…

CADx, Computer-Assisted Diagnosis.

## Discussion

Adenoma detection rate is a validated indicator of colonoscopy quality,^19^^,^^20^ with a purported 3% reduction in interval colorectal cancer prevalence for every 1% increase in ADR.^13^ Our study yielded an ADR of 36.4% for a population that includes all diagnostic, screening, and surveillance colonoscopies, well above the minimum target detection rate of 30%.^13^ A previous study conducted in this institution evaluating the performance of the same GI Genius algorithm yielded an ADR of 32.4%,^21^ thus demonstrating the potential for sustained improvement in the accuracy of deep learning algorithms over time.

Strategies including “resect-and-discard” for diminutive colorectal polyps (defined as polyps 5 mm or less in size) and “diagnose-and-leave” for diminutive rectosigmoid polyps have been endorsed by the American Society of Gastrointestinal Endoscopy Technology Committee as part of its statement on Preservation and Incorporation of Valuable Endoscopic Innovations. However, both strategies have yet to permeate into today’s standard practice owing to factors such as endoscopists’ experience and comfort, as well as limitations in current CADx technology. The “diagnose-and-leave” strategy was premised on achieving necessary thresholds, including an NPV of ≥90% for adenoma diagnosis^22^ as quoted by studies by Byrne et al.^23^ Such recommendations potentially allow for accurate identification of low-risk non-neoplastic polyps without the need for histopathological confirmation or unnecessary polypectomies, thereby reducing associated procedural risks and overall health care expenditure.

The disparity in the CADx algorithm’s NPV between colonic lesions and rectosigmoid lesions may potentially be attributed to the substantially higher disease prevalence of hyperplastic polyps within the rectosigmoid region. Although this may support the “diagnose-and-leave” strategy for diminutive rectosigmoid polyps, this may not be generalizable for polyps located in other colonic regions. Although promising, the NPV for rectosigmoid polyps achieved in this study had yet to reach recommended levels of at least 90% before “diagnose-and-leave” strategies can be confidently adopted, therefore suggesting a need for further refinement in this AI algorithm.

For example, 9 polyps that were not identified or characterized using the CADx system were decisively polypectomized by endoscopists, of which 8 returned with adenomatous histology (Table 1). This highlights the collaborative dynamic between endoscopists and AI, serving to supplement and enhance rather than replace the clinical acumen and technical skills of the endoscopist.

Endoscopists’ decision-making process often hinges on concerns about the repercussions of misdiagnoses,^17^ which invariably impact patient outcomes and carry potential medicolegal consequences for endoscopists. Patient-focused surveys similarly highlighted concerns from the patient, with up to 50% of patients citing an unwillingness to accept the “resect-and-discard” strategy and instead, keenness to bear additional health care costs for the histological analysis of all identified diminutive colorectal polyps.^24^ This being the first version of the CADx program for the GI Genius, future iterations of the program to enhance and achieve better specificity and NPV can be expected. Despite the program’s current-day limitations, it is nonetheless reassuring that nearly 90% of adenomatous polyps are being correctly identified as adenomas, encouraging endoscopists to remove them.

### Limitations and Recommendations

In total, 20.5% of polypectomies performed yielded a “no prediction” characterization. During our analysis of endoscopy videos, several common contributory factors to “no prediction” characterizations were identified. This included poor colonic mucosal exposure due to suboptimal adherence to bowel preparation. Obscuration of colonic mucosa by fecal residue may distort polyp appearance and result in a higher likelihood of non-conclusive predictions. Other technical factors included motion artifacts and inadequate time for lesion analysis before polypectomy. With limited instructional and training resources targeted at educating endoscopists on ways to fully optimize AI-assisted colonoscopy systems, the decision to take added measures to optimize bowel wall visualization largely depends on the individual endoscopist and remains difficult to standardize. Optimization of polyp surface exposure can be achieved via endoscopic maneuvers such as washing mucosal caps and retargeting the scope to achieve a stable field of view of the polyp. This does not suggest that the CADx system is incapable of analyzing polyp type on the basis of partially exposed polyp surfaces. However, optimization of polyp visualization with good endoscopic technique increases the ability of the CADx to generate conclusive and accurate polyp predictions. Malignant polyps and carcinomas established on final histology were omitted from data analysis. The GI Genius algorithm utilized in this study had been trained specifically for the identification and characterization of adenomas, which can appear morphologically similar to the naked eye. Identification of established colonic neoplasms, distinguishing between low-grade and high-grade dysplasia in adenomas, and distinguishing SSL-d from hyperplastic polyps were therefore not the intended purposes of the GI Genius. Malignant polyps and carcinomas established on final histology were thus omitted from data analysis to better reflect the premise of the GI Genius’ algorithmic training. All 16 biopsy-proven malignant lesions were identified appropriately and sampled by endoscopists, despite CADx generating non-conclusive “no prediction” characterizations. This therefore demonstrates how, ultimately, the endoscopist’s clinical acumen decision-making stills at the time of sampling remains of utmost importance and cannot be replaced with blind reliance on the current AI algorithms available.

In view of our study’s definition of “nonadenoma,” the potential for malignant lesions to be classified under this “nonadenoma” category is technically possible. A limitation of our study is therefore the reliability of the data obtained for CADx’s ability to accurately distinguish between adenomas and actual non-adenomatous lesions, especially because most colonic malignancies are adenomatous in origin on final histology. From our data, all 16 cases of biopsy-proven malignancies in our study had yielded “no prediction” characterizations, resulting in their omission from further data processing. These cases of malignant specimens were therefore not in fact erroneously classified under “nonadenoma” categories and did not have any impact on the overall predictive values calculated.

Additionally, out of the 9 polypectomies performed without preceding CADx characterization, 8 returned with adenomatous histology. Although the lack of CADx detection could have been possibly attributed to technical factors as previously raised, this emphasizes the importance of endoscopists maintaining a high index of suspicion for malignancy, where clinical acumen should not be replaced with a blind reliance on AI-generated diagnoses. Artificial intelligence guidance should instead be utilized as an adjunctive diagnostic tool to augment the physician’s decision-making process. Furthermore, AI technology provides limited benefit in the identification of established malignancies, which often display overtly suspicious morphological characteristics easily picked up by the endoscopist.

### Areas for Future Work

Sessile serrated lesions with dysplasia has a comparable risk of malignancy as with conventional tubulovillous adenomas. Due to the morphological similarity of SSLs and hyperplastic polyps, their timely identification on endoscopy poses a challenge. In our study, the incidence of SSL-d was too low for the predictive ability of the CADx algorithm to be meaningfully assessed. With future iterations of the GI Genius, the ability of the CADx algorithm to accurately identify SSL-d lesions is a worthy avenue for evaluation.

The collaborative dynamic between the endoscopist and AI is found in our study. To achieve a better illustration and assessment of this relationship, we hope to strive toward collecting more detailed and standardized data for polyp characterization in terms of qualitative characteristics, such as morphological appearance, polyp size, and endoscopist’s predicted histological result. The inclusion of the above data would enhance our ability to draw meaningful comparisons between CADx’s performance vs true polyp histology.

## Conclusion

At this current juncture, the CADx algorithm cannot yet be deemed ready to be relied on for polyp detection and characterization in mainstream health care systems. Further calibration and refinement of the CADx algorithm to improve its NPV for adenomatous polyps is undoubtedly needed before the “diagnose-and-leave” and “resect-and-discard” strategies can be accepted as a new, safe standard of care. Individual endoscopists’ skills, experience, and clinical acumen remain intangible but irreplaceable elements in the field of endoscopy that AI programs would need to surpass in order to be deemed as having superior diagnostic ability to specialists in this day and age. This, however, does not render systems like CADx useless or non-beneficial at present—such algorithms still serve as useful adjunctive diagnostic tools that may not replace expert endoscopists entirely but help by reducing mental burden and improving the overall efficiency of endoscopic procedures.

## Potential Competing Interests

Dr F.H. Koh is a Key-Opinion-Leader for Medtronic for AI in Endoscopy, Asia Pacific Region. The other authors report no competing interests.

## Ethics Statement

SingHealth Centralised Institutional Review Board reviewed the ethical proposal and waived the need for consent due to anonymised data being used.
