# Supplementary material for: Real-World Assessment of the Efficacy of Computer-Assisted Diagnosis in Colonoscopy: A Single Institution Cohort Study in Singapore
Source: Mayo Clin Proc Digit Health. 2024 Oct 26;2(4):647–55. doi: 10.1016/j.mcpdig.2024.10.002 (PMC11976013; doi:10.1016/j.mcpdig.2024.10.002)
Supplement: Supplementary Data [file mmc1.docx]

**CRediT Author Statement**

Gabrielle Koh: data collection, writing of original draft, review and editing of manuscript

Brittany Ng: data collection, writing and editing of manuscript

Frederick Koh: conceptualization, methodology, funding acquisition, overall supervision, and project administration

All other listed authors: review and editing of manuscript
